# Src family kinases-mediated negative regulation of sperm acrosome reaction in chickens (*Gallus gallus domesticus*)

**DOI:** 10.1371/journal.pone.0241181

**Published:** 2020-11-12

**Authors:** Chathura Priyadarshana, Rangga Setiawan, Atsushi Tajima, Atsushi Asano

**Affiliations:** 1 Graduate School of Life and Environmental Sciences, University of Tsukuba, Tsukuba, Ibaraki, Japan; 2 Faculty of Life and Environmental Sciences, University of Tsukuba, Tsukuba, Ibaraki, Japan; Cleveland Clinic, UNITED STATES

## Abstract

The acrosome reaction (AR) is a strictly-regulated, synchronous exocytosis that is required for sperm to penetrate ova. This all-or-nothing process occurs only once in the sperm lifecycle through a sequence of signaling pathways. Spontaneous, premature AR therefore compromises fertilization potential. Although protein kinase A (PKA) pathways play a central role in AR across species, the signaling network used for AR induction is poorly understood in birds. Mechanistic studies of mammalian sperm AR demonstrate that PKA activity is downstreamly regulated by Src family kinases (SFKs). Using SFK inhibitors, our study shows that in chicken sperm, SFKs play a role in the regulation of PKA activity and spontaneous AR without affecting motility. Furthermore, we examined the nature of SFK phosphorylation using PKA and protein tyrosine phosphatase inhibitors, which demonstrated that unlike in mammals, SFK phosphorylation in birds does not occur downstream of PKA and is primarily regulated by calcium-dependent tyrosine phosphatase activity. Functional characterization of SFKs in chicken sperm showed that SFK activation modulates the membrane potential and plays a role in inhibiting spontaneous AR. Employing biochemical isolation, we also found that membrane rafts are involved in the regulation of SFK phosphorylation. This study demonstrates a unique mechanism for regulating AR induction inherent to avian sperm that ensure fertilization potential despite prolonged storage.

## Introduction

Once ejaculated from the male reproductive tract, avian sperm undergo an acrosome reaction (AR), wherein fusion between the plasma membrane and the outer acrosomal membrane occurs [1]. AR enables sperm to release proteolytic enzymes that hydrolyze the inner perivitelline layer (IPVL) and then penetrate the oocyte [2]. Several studies focusing on signaling pathways involved in chicken sperm AR indicate both similarity to and distinction from the mechanisms underlying mammalian sperm AR [3, 4].

In mammals, sperm must undergo a maturation process called capacitation in the female reproductive tract before AR induction and successful fertilization can occur [5, 6]. Capacitation requires species-specific external stimuli in the female reproductive tract. These stimuli include lipid-binding proteins, calcium ions, and glycolyzable substrates [7]. Mammalian capacitation can also be induced by prolonged incubation with physiological stimuli and often results in spontaneous AR, i.e., premature loss of acrosome [8–10]. Although no capacitation process is recognized in avian sperm, studies from this and other groups recently showed that spontaneous AR increases when chicken sperm are incubated under mammalian capacitating conditions [3, 11]. While avian and mammalian sperm share a comparable molecular basis, unlike mammalian sperm, avian sperm spend long durations in the female reproductive tract before oocyte penetration [up to 3 weeks in chickens [12] and 2–3 months in turkeys [13]]. This suggests that there is a specific mechanism in avian sperm that sustains acrosomal responsiveness during long-term storage.

Numerous signaling pathways are involved in the increase in acrosomal responsiveness in chicken sperm. For example, calcium entry is a prerequisite for the activation of protein kinase C, calmodulin, and AMP-activated protein kinase (AMPK), which were reported to stimulate physiological AR via their respective pathways [4, 14–16]. In addition, a recent pharmacological study revealed that AMPK activity is regulated upstream by protein kinase A (PKA) [17]. It has also been documented that cAMP-dependent pathways play a central role in various signaling events in mammalian sperm, leading to capacitation-associated changes, e.g., membrane lipid remodeling, membrane hyperpolarization, elevation of intracellular Ca^2+^ ([Ca^2+^]i), and protein tyrosine phosphorylation [18]. Similarly, we recently found that cAMP-dependent pathways regulate physiological AR in chicken sperm via PKA substrate protein phosphorylation [11]; however, the coordination mechanism for how the PKA signaling network results in the elevation of acrosomal responsiveness in chicken remains unknown.

Studies on the downstream kinase of PKA in mammalian sperm demonstrated that Src family kinases (SFKs) link cAMP-dependent pathways and the capacitation-associated changes preceding AR [19–21]. SFKs are a representative group of non-receptor tyrosine kinases (c-Src, Yes, Fyn, Fgr, Lck, Hck, Blk, and Lyn), and they contribute to cell adhesion and migration [22]. In sperm, the nature of their expression and activation in response to tyrosine 416 phosphorylation differs among subtypes or species [23–25], suggesting the presence of a redundant mechanism for AR regulation. Although the regulatory mechanism for SFK activity in sperm is not understood because of these different natures, studies on immune cells [26] and discoveries related to human sperm [27] suggest that some SFK subtypes are functionally regulated by membrane rafts (MRs). MRs are functional membrane domains involved in multiple cellular processes. Our recent studies on chicken sperm revealed that these domains play a role in physiological AR induction via AMPK [16] and PKA activation [11]. However, no information is available regarding the functions of SFKs and their cross-relationship with cAMP-dependent pathways and AR induction in avian sperm. This deficit in the literature motivated us to characterize the expression and function of SFKs in chicken sperm. Our findings demonstrate that SFKs negatively regulate chicken sperm AR as an upstream modulator of PKA and suggests that membrane changes play a role in the SFK signaling pathway. This study provides new insight into the signaling regulation mechanism for AR, thereby contributing toward the prolongation of sperm fertilization potential for long-term storage in chickens.

## Materials and methods

### Reagents

All chemicals were purchased from Sigma-Aldrich (St. Louis, MO, USA) unless otherwise specified. SU6656, SKI606, and KH7 were purchased from Cayman Chemical (Ann Arbor, MI, USA). SU6656 and SKI606 are competitive inhibitors for the SFK ATP binding site, and their IC_50_ values against c-Src enzyme were 280 nM and 1.2 nM, respectively [28, 29]. Monoclonal antibodies to phospho-PKA substrate proteins, α-tubulin, and polyclonal antibodies to phospho-Src family (Tyr416) antibodies were purchased from Cell Signaling Technology (Danvers, MA, USA). Monoclonal antibodies to phospho-tyrosine and EGTA-AM were acquired from EMD Millipore (Temecula, CA, USA). MDL-12,330A HCl (MDL), 4-hydroxyphenacyl bromide [protein tyrosine phosphatase (PTP) inhibitor], and monoclonal antibodies to c-Src (H-12) were obtained from Santa Cruz Biotechnology (Dallas, TX, USA). H-89 dihydrochloride was purchased from Abcam (Cambridge, UK). Fluorescein isothiocyanate-conjugated peanut agglutinin (PNA-FITC) was obtained from J-Oil Mills, Inc. (Tokyo, Japan). Fluo 3-AM was acquired from Dojindo Laboratories (Kumamoto, Japan). DiSBAC_2_(3) was purchased from AAT Bioquest (Sunnyvale, CA, USA).

### Semen collection

Ejaculated semen was collected from at least four sexually mature Rhode Island Red roosters using the dorsal–abdominal massage method previously described [30]. Semen samples were washed in N-[Tris(hydroxymethyl)methyl]-2-aminoethanesulfonic acid (TES)-NaCl buffer (20 mM TES, 150 mM NaCl, and pH 7.4) via centrifugation at 1,000 ×g for 5 min to separate secretory fluids. All animal studies were performed with approval from the Institutional Animal Care and Use Committee of the University of Tsukuba (approval no. 18–349).

### Sperm incubation

Sperm (1 × 10^7^) were incubated for 1 h at 39°C in TES-NaCl supplemented with 2 mM Ca^2+^ and other chemicals as required for each experiment. For experiments in which the nature of the MRs was altered, 1 mM 2-hydroxypropyl-β-cyclodextrin (2-OHCD), a sterol acceptor, was used which enables disrupted MRs in chicken sperm [11].

### Evaluation of acrosome status

AR induction using IPVL, an analogue to mammalian zona pellucida, and evaluation of acrosomal status were performed as previously described [31]. In brief, sperm were centrifuged and incubated with homogenized IPVL in TES-NaCl containing 2 mM Ca^2+^ for 30 min at 39°C. The samples were centrifuged and labeled with 100 μg/ml PNA-FITC in TES-NaCl buffer for 10 min. Samples were centrifugally washed and observed under a fluorescence microscope equipped with an AF6000 imaging system (Leica Microsystems, Wetzlar, Germany). At least 200 sperm from each sample were evaluated for acrosome status. In a similar set of experiments, sperm were incubated with PNA-FITC without IPVL treatment.

### Immunoblotting

Proteins from sperm were denatured via boiling in sample buffer [32] and separated using sodium dodecyl sulfate–polyacrylamide gel electrophoresis. Transferring, blocking, and immunodetection were largely performed as previously described [33]. Dilutions for primary antibodies were 1:10000 for anti-phospho-Src family (Tyr416), anti-phospho-PKA substrate protein, anti-phospho-tyrosine, and anti-α-tubulin and 1:1000 for anti-c-Src. Secondary antibodies were diluted at 1:5000 and developed using chemiluminescence. As necessary, polyvinylidene fluoride membranes were stripped using Western blotting stripping buffer (TaKaRa Bio, Kusatsu, Japan), and reprobed for others. Resulting bands were subjected to densitometry using ImageJ software (version 1.51). α-tubulin was used as a loading control.

### Measurement of [Ca^2+^]i level

Sperm were treated with 5 μM Fluo 3-AM in TES-NaCl containing 2 mM Ca^2+^ for 30 min and centrifugally washed to remove any extracellular dye [11]. Sperm were incubated with respective chemicals (SKI606, SU6656, H89, or combinations of these) for 30 min. As a positive control, 1 μM calcium ionophore A23187 was added to the sperm suspensions. Fluorescence intensity was measured using a DTX 800 spectrophotometer (Molecular Devices, Sunnyvale, CA, USA) with a 485 nm excitation filter and a 535 nm emission filter. No difference was observed in fluorescence intensity due to the type and concentration of the inhibitors.

### Preparation of Detergent-Resistant Membrane (DRM)

MRs were isolated from sperm as low density DRMs as previously described [34]. In brief, sperm membranes were isolated by dounce homogenization and sonication in phosphate-buffered saline (PBS) containing a protease inhibitor cocktail (Roche Diagnostics, Mannheim, Germany) and centrifuged at 10,000 ×g for 10 min. The resultant supernatants were further centrifuged at 20,000 ×g for 2 h to obtain membrane fractions. Samples were incubated with 0.5% Triton X-100 (TX-100) in PBS for 30 min at 4°C and then centrifuged at 20,000 ×g for 2 h to separate the DRM and soluble fraction, which mostly comprised non-raft domains.

### Membrane potential assay

Sperm were incubated with 0, 0.1, 1, 10, or 100 μM SFK inhibitors (SKI606 or SU6656) in TES-NaCl containing 2 mM Ca^2+^ for 30 min. They were further incubated for 5 min after the addition of 1 μM membrane potential probe DiSBAC_2_(3) as previuosly described [35]. No mitochondrial uncouplers were used because their contribution to the resting potential had been determined to be insignificant [36]. Fluorescence intensities in sperm were measured using a DTX 800 spectrophotometer (Molecular Devices) with a 535/560 nm excitation/emission wavelength.

Calibration was performed by adding 1 μM gramicidin and sequential additions of 1 M KCl solution to the medium (20 mM TES, 150 mM choline chloride, and pH 7.4) in which Na^+^ was equally replaced with choline^+^. This medium allows K^+^ to be the only external ion capable of permeating the gramicidin pore [36]. The initial potassium concentration was 5.2 mM KCl; additional amounts of KCl were added to the final concentrations of 8, 12, 25, 40, and 66 mM KCl, corresponding to plasma *Em* of −80, −69, −59, −40, −28, and -15 mV, respectively. These values were obtained using the Nernst equation, assuming a [K^+^]*i* of 120 mM [37]. The final sperm membrane potential was obtained by linearly interpolating theoretical Em values against arbitrary fluorescence units of each trace (S1 Fig). DiSBAC_2_(3) has been successfully used in mammalian sperm, and it has no toxic effects on sperm function at the concentrations used (<5 μM) [36].

### Immunocytochemistry

Sperm were fixed with 4% paraformaldehyde, permeabilized with 0.5% TX-100, and blocked with 10% goat serum for 1 h. Samples were incubated with or without anti-c-Src, anti-phospho-Src family (1:200) or normal rabbit IgG (R & D Systems, Minneapolis, MN, USA) antibodies in PBS overnight at 4°C. Sperm were incubated with anti-mouse immunoglobulin G (IgG) conjugated with Texas Red (1:200) and 100 μg/mL PNA-FITC or anti-rabbit IgG conjugated with Alexa Fluor 594 (1:400) for 1 h. Coverslips were mounted using VECTASHIELD mounting medium with DAPI (Vector Laboratories, Burlingame, CA, USA).

### Motility assay

Sperm were treated with or without 10 μM SKI606 and SU6656 in TES-NaCl containing 2 mM Ca^2+^ for 1 h at 39°C and were evaluated for their motility profile using the sperm motility analysis system (SMAS, DITECT, Tokyo, Japan). The parameters measured were proportion of motile spermatozoa, progressive velocity (VSL; straight line between the beginning and end of the track/time elapsed), curvilinear velocity (VCL; average velocity measured over the actual path), path velocity (VAP; velocity/average position of the spermatozoa), linearity (LIN; departure of the cell track from a straight line; LIN = [VSL/VCL]), straightness (STR; STR = [VSL/VAP]), amplitude of lateral head displacement (ALH), and beat-cross frequency.

### Data analyses

Multiple comparisons were performed using Tukey’s honest significant difference test. Results are expressed as mean ± standard error of the mean (SEM). Probability values < 0.05 were considered significant.

## Results

### Expression and localization of c-Src and p-SFK-Y416

**Fig 1:**
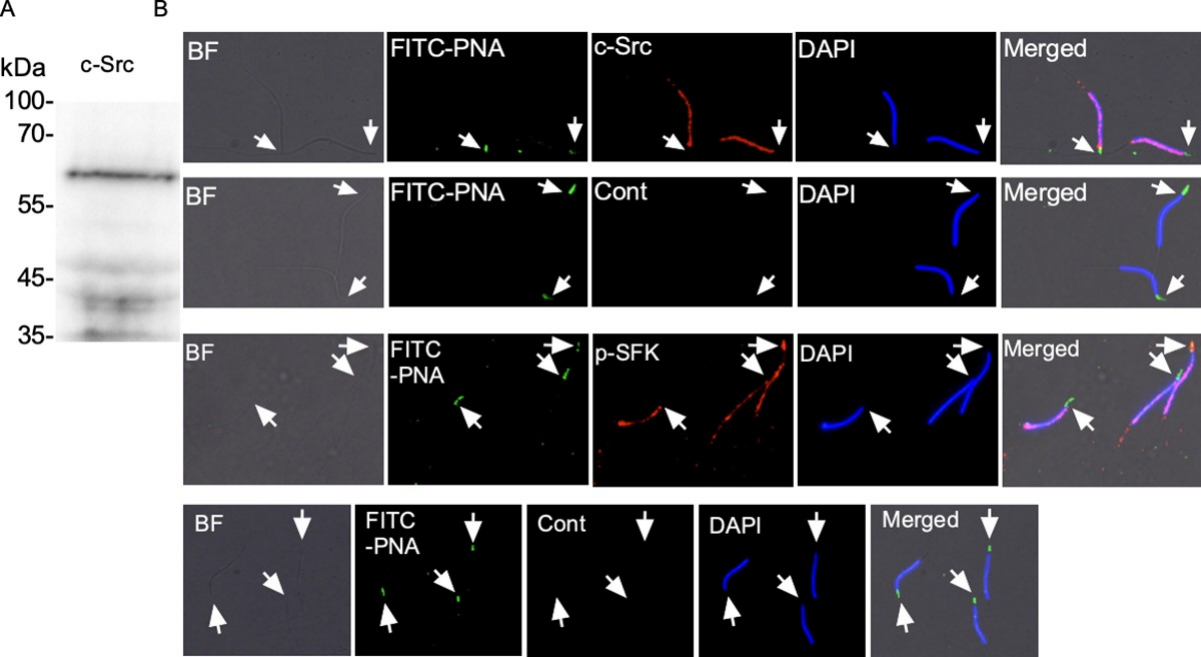
Localization of c-Src and p-SFK-Y416 in chicken sperm. c-Src was immunodetected at a predicted molecular weight (A). Sperm acrosomes (arrows) were stained by FITC-PNA (B and C). c-Src was localized to the sperm head except for the acrosomal region (B). Similarly, p-SFK-Y416 was localized to the sperm head (C). Acrosomal localization was seen in some sperm. No signals were observed in control sperm (B and C). Images are representative of three replicate trials.

Immunoblotting analysis of chicken sperm showed the presence of c-Src at a predicted molecular weight (60 kDa; Fig 1A). FITC-PNA was used to localize the sperm acrosomes (arrows). c-Src was localized to the sperm head, except for the acrosomal region (Fig 1B). The tyrosine 416 phosphorylated form of SFK (p-SFK-Y416) localization was also observed in the head region, and the acrosomal region in certain sperm (Fig 1C). No signals were observed in the control sperm treated with normal rabbit IgG control instead of those antibodies (Fig 1B and 1C). These results suggest that c-Src and p-SFK-Y416 were localized to the sperm head and the acrosomal region.

### Involvement of SFK phosphorylation in regulation of PKA activity

**Fig 2:**
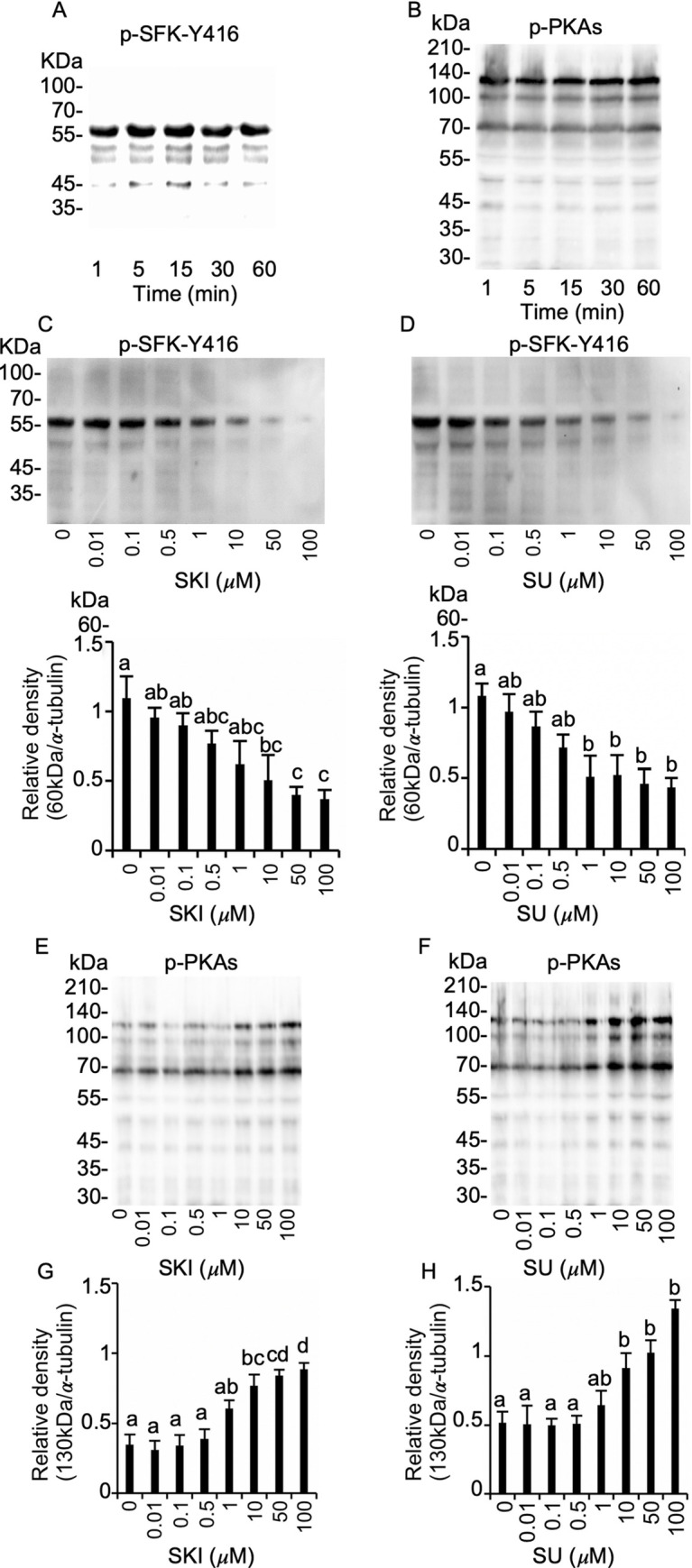
Regulation of PKA substrate protein phosphorylation by SFKs in chicken sperm. Sperm were incubated in TES-NaCl + 2mM Ca^2+^ for 1 hour with 0–100 μM SFK inhibitors (SKI606 and SU6656) and then subjected to immunoblotting to obtain a phosphorylation profile of SFK-Y416 and PKA substrate proteins. No change was observed in p-SFK-Y416 (A) and p-PKA substrate protein (B) in the incubation period. Downregulation of p-SFK-Y416 in response to SFK inhibition by SKI606 (C) and SU6656 (D). SKI606 and SU6656 reduced p-SFK-Y416 (C and D) but increased p-PKA substrate proteins (E, F, G, and H). All immunoblotting analyses were repeated at least four times. Data are presented as mean ± SEM. ^ad^*P* < 0.05.

Chicken sperm were incubated for 1, 5, 15, 30, and 60 min and immunoblotted for the presence of p-SFK-Y416. p-SFK-Y416 was detected at the predicted molecular weight (60 kDa) and did not differ significantly among the incubation periods (Fig 2A), suggesting that autophosphorylation was not involved. Similarly, no change was observed in the expression of phosphorylated PKA substrate protein (p-PKAs) (Fig 2B). To validate a role of SFK in PKA signaling cascade, we used SKI606 and SU6656 which differ in inhibitory spectra against SFKs. Incubation of sperm with 0–100 μM SKI606 and SU6656 resulted in dose-dependent inhibition of p-SFK-Y416 expression. Densitometry analyses showed significant reduction at ≥10 μM SKI606 and ≥1 μM SU6656 (Fig 2C and 2D). In contrast, at the same concentrations, these SFK inhibitors increased p-PKAs in a dose-dependent manner, with significant differences at ≥10 μM for both inhibitors (Fig 2E–2H). No difference in protein tyrosine phosphorylation was observed (S1A–S1D Fig). These results suggest that SFK de-phosphorylation upregulates PKA activity.

### SFK phosphorylation is not a downstream PKA event

**Fig 3:**
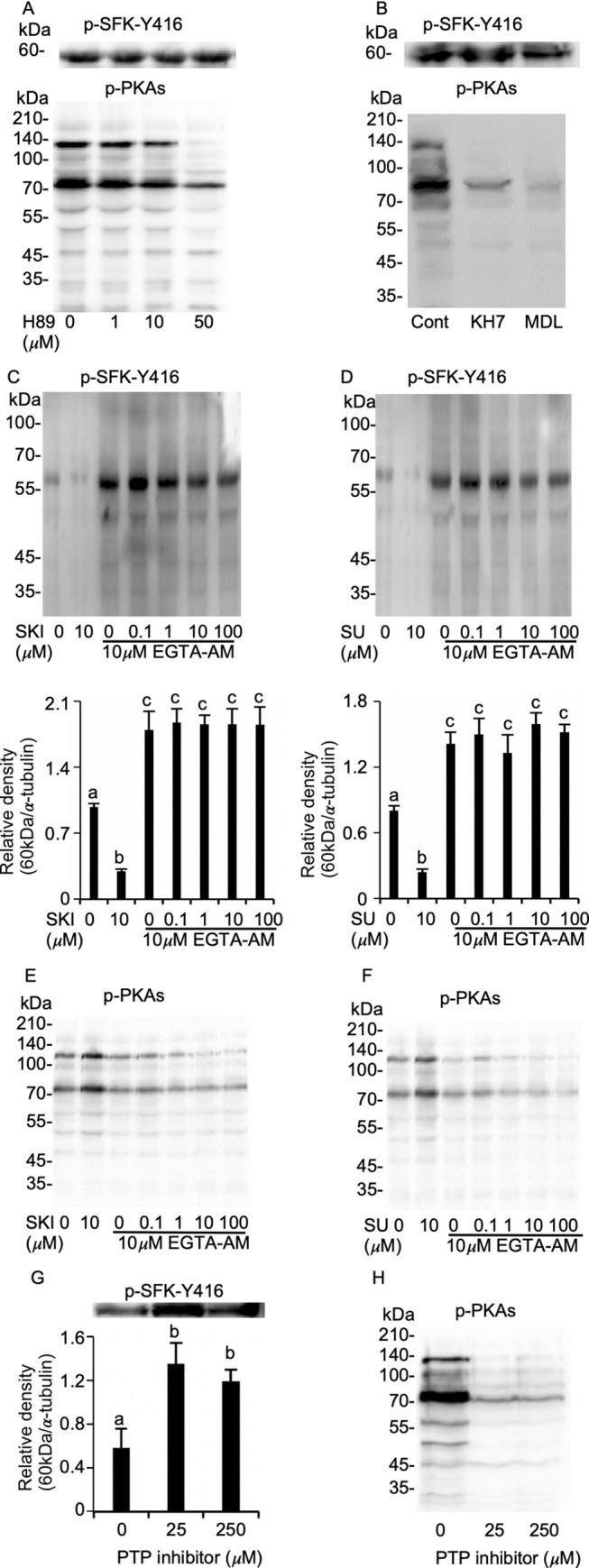
Mechanism of SFK phosphorylation and negative regulation of PKA in chicken sperm. Sperm were incubated with 0–50 μM H-89 (PKA inhibitor), 25 μM KH7 (sAC inhibitor), 50 μM MDL (tmAC inhibitor), 0–250 μM PTP inhibitor, or EGTA-AM in the presence or absence of SKI606 and SU6656 and then subjected to immunodetection for p-SFK-Y416 or p-PKAs. Treatment with H89, KH7, and MDL did not change p-SFK-Y416 despite the reduction of p-PKAs (A and B). EGTA-AM treatment increased p-SFK-Y416 regardless of the presence of SKI606 and SU6656 (C and D), concomitant with abrogated increases in p-PKAs by SFK inhibition (E and F). Treatment with PTP inhibitor concurrently increased p-SFK-Y416 and decreased p-PKAs (G and H). All immunoblotting analyses are representative of four replicate trials. Data are presented as mean ± SEM. ^ab^*P* < 0.05.

We found that in chicken sperm, inhibition of PKA activity by H89 did not affect p-SFK-Y416, whereas the amount of p-PKAs dramatically decreased (Fig 3A). Similarly, p-PKAs alone reduced when soluble and transmembrane adenylyl cyclase (sAC and tmAC) were inhibited by KH7 and MDL (Fig 3B), suggesting that SFK phosphorylation is not the downstream of PKA event. To identify the regulatory mechanism involved in SFK phosphorylation, the relationship between p-SFK-Y416 level and [Ca^2+^]i was examined by incubating sperm for 45 min under SKI606 or SU6656 concentrations after preloading with 10 μM EGTA-AM for 15 min. This showed a dramatic increase in p-SFK-Y416 level regardless of the presence or absence of SFK inhibitors (Fig 3C and 3D). Concomitantly, an increased p-PKAs in response to SFK inhibition was negated by calcium chelation (Fig 3E and 3F) despite of no change by EGTA-AM alone (S3 Fig), indicating the negative regulation of calcium in SFK-Y416 phosphorylation. PTP, which removes phosphate from phosphorylated tyrosine residues of proteins, functionally contributes to mammalian sperm AR. Therefore, the role of PTP in SFK-Y416 phosphorylation was examined in sperm treated with PTP inhibitor with high affinity to SHP-1 and PTP 1B of calcium-dependent phosphatases, indicating that p-SFK-Y416 increased and p-PKAs decreased (Fig 3H and 3I). These results suggest the regulatory role of PTP in SFK phosphorylation. Collectively, these results suggest that SFK phosphorylation occurs upstream of PKA signaling pathway in chicken sperm, possibly through a calcium-dependent phosphatases.

### SFK inhibition triggers spontaneous AR via membrane potential hyperpolarization

**Fig 4:**
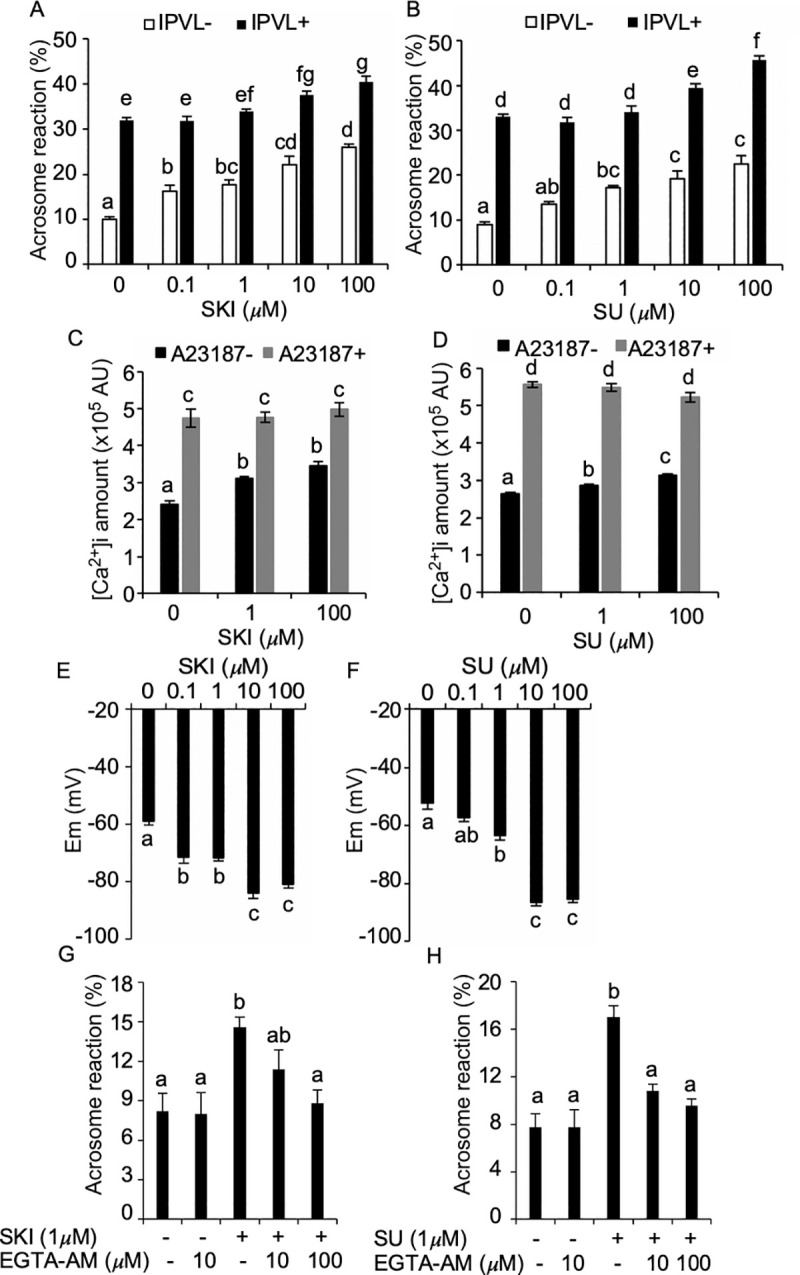
The roles of SFK in the regulation of chicken sperm AR. Sperm were incubated in TES-NaCl + 2mM Ca^2+^ with 0–100 μM SKI606 and SU6656 for 1 h, and subjected to evaluation of spontaneous and physiological AR (IPVL− and IPVL+). Both SFK inhibitors stimulated both AR types in a dose-dependent manner (A and B). Fluo 3-AM loaded sperm were treated with SFK inhibitors and subjected to [Ca^2+^]i measurements. [Ca^2+^]i increased in response to SKI606 (C) and SU6656 (D) treatments. Sperm were incubated with 0–100 μM SFK inhibitors and then treated with DiSBAC_2_(3) for membrane polarization assay. Both SKI606 and SU6656 treatment induced hyperpolarization potential (E and F) representive from a dose-dependent reduction of fluroscent intensity. Sperm were loaded with 0, 10, or 100 μM EGTA-AM, incubated for 45 min with or without 1 μM SFK inhibitors, and examined with spontaneous AR. EGTA-AM treatment abrogated both SKI606- and SU6656-stimulated spontaneous AR (G and H). Data are presented as mean ± SEM. ^ag^*P* < 0.05.

Sperm were incubated with 0–100 μM SKI606 or SU6656 and subjected to AR assay, [Ca^2+^]i measurement, or membrane potential assay. The incubation of sperm with SKI606 increased acrosome-reacted sperm in dose-dependent manner without IPVL stimulation (10.0% ± 0.6%, 16.3% ± 1.3%, 17.8% ± 1.0%, 22.3 ± 1.8%, and 26.0 ± 0.7%, respectively) (Fig 4A). IPVL stimulation significantly increased AR in sperm treated with SKI606 concentrations, which resulted in the dose-dependent elevation of acrosome-reacted sperm (32.0% ± 0.6%, 31.8% ± 1.0%, 34.0% ± 0.4%, 37.5% ± 0.9%, and 40.5% ± 1.2%, respectively). Similarly, spontaneous AR were increased in a dose-dependent manner when treated with SU6656 (9.0% ± 0.6%, 13.5% ± 0.6%, 17.3% ± 0.5%, 19.3% ± 1.8%, and 22.5% ± 1.8%; 33.0% ± 0.6%, 31.8% ± 1.1%, 34.0% ± 1.5%, 39.5% ± 1.0%, and 45.8% ± 0.9%) (Fig 4B). Furthermore, we evaluated net IPVL-induced AR, based on both spontaneous AR and total AR, which showed the reduction under SKI606 supplementation (S4 Fig). Altogether, these results show that SFK inhibition induces spontaneous AR and thereby increases total AR sperm following IPVL stimulation. There was no significant difference in motility parameters of sperm treated with 10 μM SFK inhibitors, suggesting that the inhibitors had no adverse effect on sperm (S1 Table).

To validate the relationship between [Ca^2+^]i and spontaneous AR induction, we examined the change in [Ca^2+^]i following incubation with 0, 1, or 100 μM SFK inhibitors of sperm preloaded with a fluorescent calcium indicator dye Fluo 3-AM, and found dose-dependent increases in [Ca^2+^]i in response to SKI606 and SU6656 treatments (Fig 4C and 4D). Changes as per the time-course change in [Ca^2+^]i were shown in S5 Fig.

To validate the relationship between SFKs and membrane potential, we examined changes in the membrane potential in response to SFK inhibition, using DiSBAC_2_(3). The membrane potential assay showed that a hyperpolarizing current occurred in response to SKI606 and SU6656 used at ≥0.1 μM and 1 μM (Fig 4E and 4F). To examine the role of [Ca^2+^]i in spontaneous AR, acrosomal status was examined in EGTA-AM preloaded sperm, and then treated with SKI606 or SU6656. This showed that calcium chelating inhibited spontaneous AR that were increased by SFK inhibition (Fig 4G and 4F). Taken together, our results suggest that SFK inhibition induced membrane hyperpolarization, thereby stimulating [Ca^2+^]i influx, which results in spontaneous AR.

**Fig 5:**
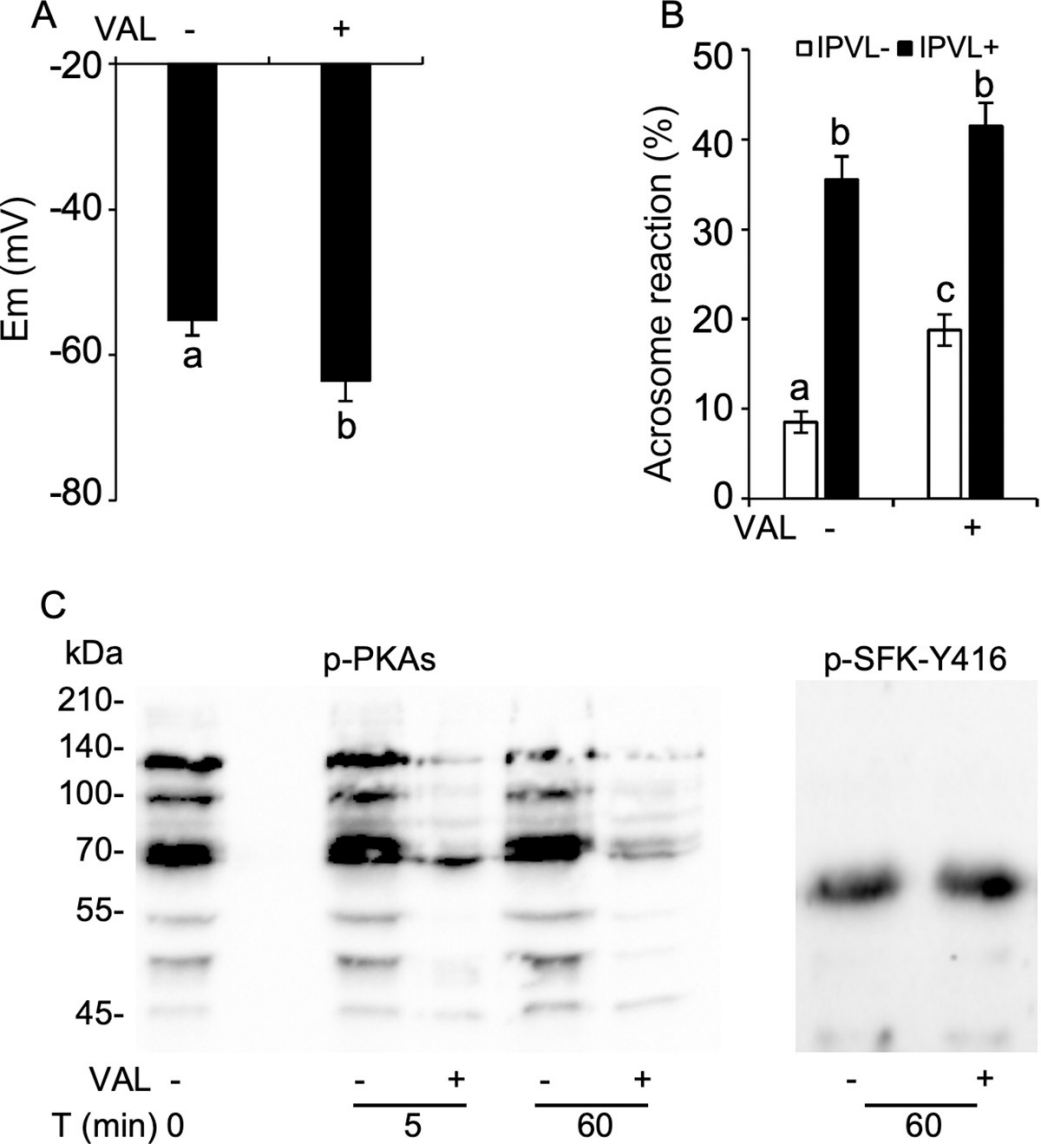
The relationship between membrane hyperpolarization and spontaneous AR. Sperm were incubated with 0 or 1 μM valinomycin, a K^+^ ionophore, for 60 min in TES-NaCl + 2mM Ca^2+^, and subjected to membrane polarization assay or evaluation of spontaneous and IPVL-induced AR. Valinomycin treatment induced hyperpolarizing potential (A) and elevation of spontaneous AR (B). Sperm were incubated for 5 and 60 min with 0 or 1 μM valinomycin. p-PKAs reduced in response to valinomycin treatment despite of no change in p-SFKs (C). All immunoblotting analyses are performed in triplicates. Data are presented as mean ± SEM. ^ac^*P* < 0.05.

To validate the relationship between membrane hyperpolarization and spontaneous AR, we examined spontaneous AR in sperm incubated for 1 h with 1 μM valinomycin, which is a potassium ionophore. Valinomycin treatment induced hyperpolarized potential (Fig 5A) and it also increased spontaneous AR (18.8% ± 1.8%) as compared with control sperm (8.5% ± 1.2%). We found that the total AR increased in response to IPVL stimulation in control and valinomycin-treated sperm (35.5% ± 2.6% and 41.5% ± 2.6%) (Fig 5B). Net IPVL-induced AR did not differ between treatments (Control: 27.9% ± 1.7%, Valinomycin: 22.8% ± 1.3%) (S6 Fig). These results corroborate the role of membrane hyperpolarization in spontaneous AR. Surprisingly, p-PKAs were reduced in response to valinomycin treatment despite of no change in p-SFKs (Fig 5C), suggesting that membrane hyperpolarization connects between SFK and PKA with a primary role in spontaneous AR.

### Involvement of MRs in SFK phosphorylation

**Fig 6:**
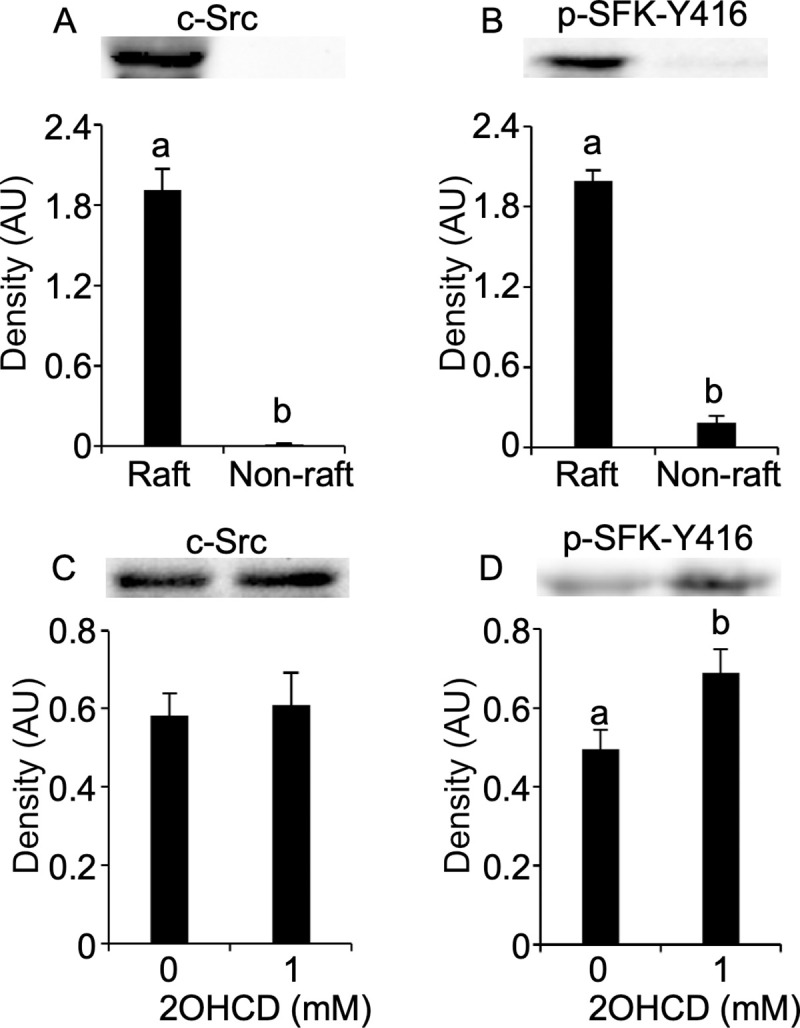
Membrane rafts-mediated regulation of SFK phosphorylation. Sperm membranes were separated into insoluble and soluble membranes (Raft and non-rafts) to TX-100 treatment. Immunoblots for p-SFK-Y416 and c-Src revealed their association with membrane rafts (A and B). Sperm were incubated with 0 or 1 mM 2-OHCD, and then immunoblotted for c-Src and p-SFK-Y416. Increases in p-SFK-Y416 were observed in response to 1 mM 2-OHCD treatment (D) despite no differences in c-Src (C). All immunoblotting analyses are representative of four replicate trials. Data are presented as mean ± SEM. ^ab^*P* < 0.05.

To evaluate the functional linkage of SFKs to MRs, we isolated detergent-insoluble membranes composed of MRs and detergent-soluble membranes composed of non-MRs, and examined the association of SFK phosphorylation with MRs. c-Src, an SFK member, and p-SFK-Y416 were exclusively enriched in MRs (Fig 6A and 6B), suggesting their association with MRs. This led us to evaluate the change in p-SFK-Y416 in response to treatment with 1 mM 2-OHCD, a cholesterol acceptor, to disrupt the MRs [11]. 2-OHCD treatment increased p-SFK-Y416 expression (Fig 6D), while there was no difference in c-Src expression (Fig 6C). These results suggest biochemical and functional association of SFK phosphorylation with MRs.

## Discussion

**Fig 7:**
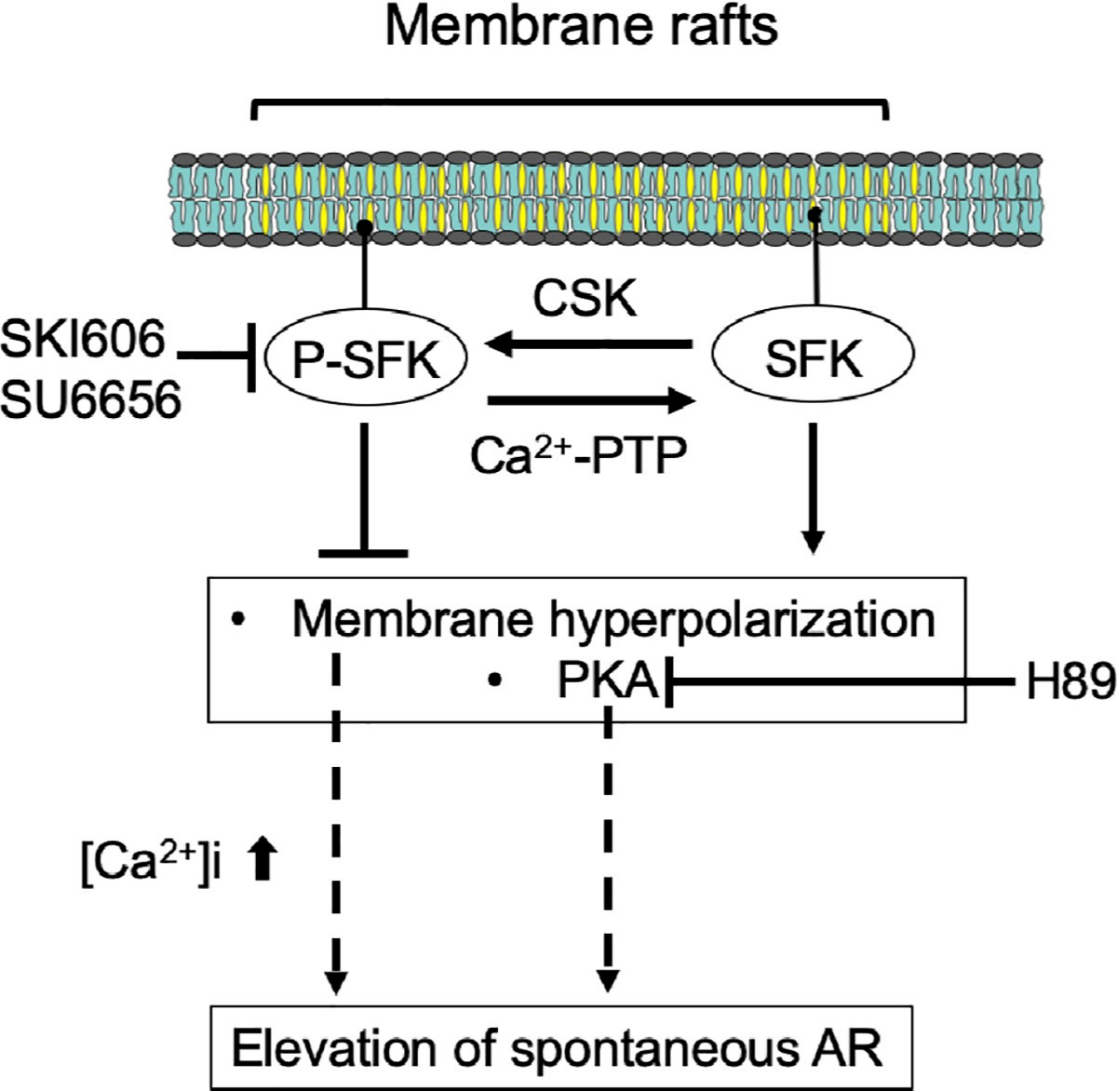
Schematic model of SFK-mediated regulation of sperm AR via PKA pathway. The plasma membrane of sperm contains multiple membrane rafts where SFK are spatially and functionally associated. SFK phosphorylation/dephosphorylation are regulated by an equilibrium between C-terminal Src kinase (CSK) and Ca^2+^-dependent PTP. SFK phosphorylation/dephosphorylation is involved in the regulation of PKA activity and spontaneous AR through modulation of membrane potential.

We previously demonstrated that AR is regulated via cAMP-dependent pathways in avian sperm [11]; however, the regulation mechanism of acrosomal responsiveness by PKA activation was not fully understood. In mammals, activation of this kinase mediates SFK-Y416 phosphorylation, resulting in hyperactivated motility and elevated acrosomal responsiveness; however, the identity and roles of SFK remained unknown in avian sperm. Our results demonstrate that SFK phosphorylation plays an important role in acrosomal responsiveness via regulation of PKA activity and membrane potential in chicken sperm. Combining the results from this study and other studies that have identified the nature of SFK in sperm [20, 21, 38], we have generated a working model with regard to SFK-mediated regulation of acrosomal responsiveness (Fig 7). The plasma membrane of the overlaying sperm head region contains multiple MRs where SFK are spatially and functionally associated. SFK phosphorylation/dephosphorylation are regulated by the equilibrium between C-terminal Src kinase (CSK) [23] and a Ca^2+^-dependent PTP such as PTP1B [39, 40]. SFK phosphorylation contributes to not only negative regulation of PKA activity, but also prevent spontaneous AR by membrane hyperpolarization that contributes to ion influx/efflux, such as potassium and calcium. Altogether, our results provide novel insights into the membrane regulation of signal transduction pathways causing AR in chicken sperm and also highlight a distinction in the AR mechanisms of avian and mammalian sperm.

The presence of SFKs have been reported in the sperm of a few species [24, 25]. Our immunoblotting analysis showed the presence of c-Src, a prototypic member of SFK, in addition to p-SFK-Y416, in chicken sperm. p-SFK-Y416 was present in patch-like patterns over the sperm head, including the acrosomal region, which differed from the c-Src only enriched in the sperm head except for acrosomal region. This suggests that the primary SFK being phosphorylated is not c-Src, which is consistent with previous results showing a species-specific preference of phosphorylated SFK members in mammalian sperm [24]. Further research is required to identify the SFKs in chicken sperm.

Previous immunoblot analyses regarding mammalian sperm demonstrated that SFK activation characterized by p-SFK-Y416 accumulation occurs when sperm were incubated *in vitro* under capacitating conditions, including both HCO_3_^-^ and Ca^2+^ [23]. In contrast, our results revealed high expression of p-SFK-Y416 without any stimulative treatment, suggesting a mechanistic distinction in SFK activation for mammals and birds. Using SFK-specific inhibitors SKI606 and SU6656, previous studies demonstrated that phosphorylation of SFK-Y416 occurs soon after PKA activation in response to the elevation of cytoplasmic cAMP content in mammalian sperm, resulting in the elevation of protein tyrosine phosphorylation necessary for physiological AR and hyperactivated motility [16, 32]. In comparison, our results showed contrasting results, as SKI606 and SU6656 treatment not only decreased p-SFK-Y416 in a dose-dependent manner but also increased PKA activity at ≥10 μM, a similar concentration that inhibited SFK and PKA activity in several types of mammalian sperm [21, 38]. Because these are competitive inhibitors for SFK ATP binding sites with difference in inhibitory spectrum between SFK subtypes [28, 29], we carried out SMAS analysis, which showed no adverse effect on flagellar motility. These results collectively suggest that SFK activation negatively regulates PKA activity in chicken sperm. It is currently unknown whether the reduction of p-PKAs was mediated by direct or indirect effects of SFK phosphorylation.

In mammalian sperm, SFK activation is a downstream event of PKA activation [20, 21, 38]. However, our results show that none of the PKA inhibitors with varied actions inhibited SFK phosphorylation, suggesting that SFK phosphorylation is not a downstream event of PKA activation, at least in chicken sperm. *In vitro* kinase assays in human sperm revealed that SFK activity is potentiated in the presence of calcium [23], suggesting the involvement of calcium in upstream of SFK activation. Therefore, we tested this possibility, and found that [Ca^2+^]i inhibits p-SFK-Y416. SFK activity is modulated by molecular interactions that depend on an equilibrium between tyrosine phosphorylation and dephosphorylation of catalytic sites [41]. This led us to test the possible role of PTP on p-SFK-Y416 and PKA activity. Surprisingly, we found that while using a specific inhibitor, PTP inhibition increased drastically in p-SFK-Y416 concomitant with decreases in PKA activity. Together this suggests that calcium-dependent PTP is involved in the regulation of p-SFK-Y416. In fact, murine and human sperm contains highly active tyrosine phosphatases that play a role in the regulation of acrosomal responsiveness [42]. For example, a study using obese mice demonstrated that excess activation of PTP1B, a PTP sub-type, impairs AR, resulting in infertile phenotypes [39]. In other cells, PTP1B activity is regulated by calpain in calcium-dependent manner [43] and is responsible for SFK activation [44]. Altogether with previous results that suggest calpain activation is involved in the AR of mammalian sperm [45, 46], and that chicken sperm possesses calpain subtypes [47], it is probable that PTP plays a role in the regulation of SFK activity.

Interestingly, we demonstrated that SFK inhibition dose-dependently stimulated spontaneous AR, resulting in an elevated overall percentage of AR induction after physiological stimulation. This is opposed to murine results that suggest SFK activation is a prerequisite for capacitation-associated changes, including PKA activation, and elevated acrosome responsiveness. Our result is instead in agreement with boar and bull sperm studies which demonstrated that SFK inhibition by SU6656 stimulated spontaneous AR via calcium-dependent pathways, such as actin depolymerization [48, 49]. Although the mechanism of acceleration of spontaneous AR is not fully understood, lines of study in mammalian sperm suggested the possible involvement of [Ca^2+^]i increases in spontaneous AR [49–52]. In agreement with these, our results showed that SFK inhibition was accompanied with [Ca^2+^]i increases. SFKs have been known to regulate a variety of membrane ion channels such as ligand-, voltage-, and second messenger-gated channels [53]. In murine sperm, membrane hyperpolarization is a prerequisite for [Ca^2+^]i increase that enables sperm to undergo AR [54]. Therefore, we conducted membrane potential assays using a fluorescent potential probe, which showed that SFK inhibition induced hyperpolarizing potential in a dose-dependent manner. Although the mechanism responsible for membrane hyperpolarization by SFKs in chicken sperm remains unclear, previous studies using heterologous expression systems combined with pharmacological murine sperm experiments suggested that potassium efflux occurs via SLO3, a sperm-specific potassium channel, in response to SFK activation, which eventually stimulates membrane hyperpolarization preceding a series of capacitation processes [25]. Despite the fact that capacitation processes have not been recognized in birds, reptiles, and fish, SLO3 is predominantly expressed in their testes, accordant to mammalian SLO3 [55]. Our results suggest investigation for the relationship among chicken SFK, SLO3, and membrane potential, using heterologous expression system.

Studies using murine sperm showed that hyperpolarization is insufficient to induce capacitation associated changes including protein phosphorylation events, but allows sperm to undergo AR in response to physiological stimulation [25, 54]. Using valinomycin, a potassium ionophore that hyperpolarizes murine sperm [56], we found valinomycin-induced hyperpolarizing current as well as elevated spontaneous AR in chicken sperm. Although this is in agreement with our view on the induction of spontaneous AR in response to SFK inhibition, it was surprising that valinomycin reduced p-PKAs, which is in contrast to the aforementioned result on the consequence of SFK inhibition. Although it is difficult to clarify this mechanism yet, one possibility is that it may result from the differences in nature between valinomycin- and SFK inhibition-induced hyperpolarization. This is because 10 μM SKI606 and SU6656 treatments hyperpolarized *Em* by approximately −85 mV, whereas 1 μM valinomycin, same concentration as usually used for mammalian sperm, only did by approximately −63 mV, which indicates differences in ion channels and transporters with voltage-dependence. Further studies are required to determine the mechanistic nature of membrane hyperpolarization in chicken sperm.

Studies in other cells demonstrated the functional and spatial associations of SFKs, with MRs playing significant roles in an assortment of cellular processes [57]. In addition to previous findings that MRs are present in mammalian sperm and regulate multi-stage fertilization [58, 59], our results have revealed that chicken sperm possess membrane domains that play an important role in AR induction and binding to IPVL [11, 16, 60]. This study found a relative abundance of c-Src and p-SFK-Y416 in TX-100 insoluble membrane fractions that compositionally mimic MRs, which was different from previous results in which bull sperm have more enriched p-SFK-Y416 in non-rafts than MRs [61]. This suggests that unlike mammalian sperm, SFK signaling modules may have a high affinity to MRs in chicken sperm. The functional association was confirmed by evidence that p-SFK-Y416 increased in response to 2OHCD treatments that were previously shown to sufficiently induce MR disruption [11]. This is in agreement with a previous study using cultured cells wherein disruption of MRs by sterol removal increased both SFK and its substrate protein phosphorylation [62]. Although few studies have detailed how sterol efflux results in SFK activation in chicken sperm, this could be attributed to the enhanced dissociation of CSK from MRs. CSK is a negative regulator of p-SFK-Y416, and known to be recruited into MRs, where it interacts with SFKs [63]. Sterol removal was found to disassemble this interaction, which causes spatial shift of SFKs to out of the MR, leading to their activation [62]. Considering with the presence of CSK in mammalian sperm [20, 23], our results suggest deeper examinations on the membrane regulation of SFK activity in chicken sperm.

Despite the commonality of functional connections between membrane hyperpolarization and AR induction between species, our results suggest that in chicken sperm, SFK activation acts contrary to those in murine and human sperm, inducing suppressive effects on spontaneous AR without affecting acrosomal responsiveness to physiological stimulation. Spontaneous AR often take place during capacitation periods in mammals [64] and are currently viewed as a type of sperm malfunction that induces the premature loss of fertilization ability [65]. Similar to birds, urodele amphibians typically store sperm in the vas deferens and cloacal glands to use them for insemination after hibernation. A recent study showed that sperm storage in the vas deferens increases spontaneous AR potential by PKA activation and [Ca^2+^]i increases [66]. Considering that chicken sperm is able to reside in sperm storage sites in the female tract for up to 2–3 weeks [67], our results provide a hypothesis that regulation of PKA and membrane potential by SFK activation play a role in sustaining fertilization potential by suppressing spontaneous AR during storage periods and in ensuring populations of sperm that are able to undergo AR in response to physiological stimulation.

In summary, this study demonstrated that SFK phosphorylation plays an important role in acrosomal responsiveness via regulation of PKA and [Ca^2+^]i levels. Despite the common nature of mammalian and avian sperm, our results showed that, unlike in mammals, SFK activation in birds occurs upstream of PKA-dependent protein phosphorylation and is supported by signaling modules associated with MRs, suggesting a possible mechanism for regulating acrosomal responsiveness and enabling them to sustain fertilizing potential during prolonged storage.

## Supporting information

S1 Fig Membrane polarization assay. Sperm were incubated with 0–100 μM SFK inhibitors and then treated with DiSBAC_2_(3) for membrane polarization assays via measuring fluorescent intensity. Calibration was performed by adding 1 μM gramicidin and sequential additions of 1 M KCl solution to the medium (20 mM TES, 150 mM choline chloride, and pH 7.4), in which Na^+^ was replaced with choline^+^ at equal concentration. The initial potassium concentration was 5.2 mM KCl; additional amounts of KCl were added to the final concentration of 8, 12, 25, 40, and 66 mM KCl, corresponding to plasma *Em* of −80, −69, −59, −40, −28, and −15 mV, respectively, based on the Nernst equation (n = 8).
(TIF)

S2 Fig Protein tyrosine phosphorylation in chicken sperm incubated with SFK inhibitors. Sperm were incubated with 0–100 μM SKI606 or SU6656 and then subjected to immunoblotting. No difference was detected in p-tyrosine profile regardless of SFK inhibition by SKI606 (A and B). This is consistent with densitometry data (C and D). All immunoblotting analyses are representative of four replicate trials. Data are presented as mean ± SEM.
(TIF)

S3 Fig Densitometric analysis corresponding to Fig 3E and 3F. Sperm were preloaded with EGTA-AM for 15 min, and incubated under respective condition for 45 min. ^ab^*P* < 0.05.
(TIF)

S4 Fig Effect of SFK inhibitors on IPVL-induced AR. Net % of IPVL was obtained by subtracting of % spontaneous AR from total AR % after IPVL treatment. ^ab^*P* < 0.05.
(TIF)

S5 Fig Time-course change in [Ca^2+^]i. Sperm were loaded with Fluo 3-AM for 30 min. Fluorescence intensity of Fluo 3 was measured at 5, 15, and 30 min after incubation under the presence of 0, 1, and 100 μM SKI606 (SKI) or SU6656 (SU). Sperm were treated with 1 μM calcium ionophore as a positive control. ^ad^*P* < 0.05 in the same time point. ^AC^*P* < 0.05 in the same treatment.
(TIF)

S6 Fig Effects of valinomycin treatment on net % of IPVL-induced AR. (TIF)

S1 Table Sperm motility profile. Sperm were incubated with or without 10 μM SKI606 and SU6656 and were subjected to sperm motility analysis. No differences were observed in motility parameters across treatments. Data are presented as mean ± SEM (n = 4).
(TIF)

S1 Raw images (PDF)
