# Supplementary figures and images for: Src family kinases-mediated negative regulation of sperm acrosome reaction in chickens (Gallus gallus domesticus)
Source: PLoS One. 2020 Nov 12;15(11):e0241181. doi: 10.1371/journal.pone.0241181 (PMC7660528; doi:10.1371/journal.pone.0241181)

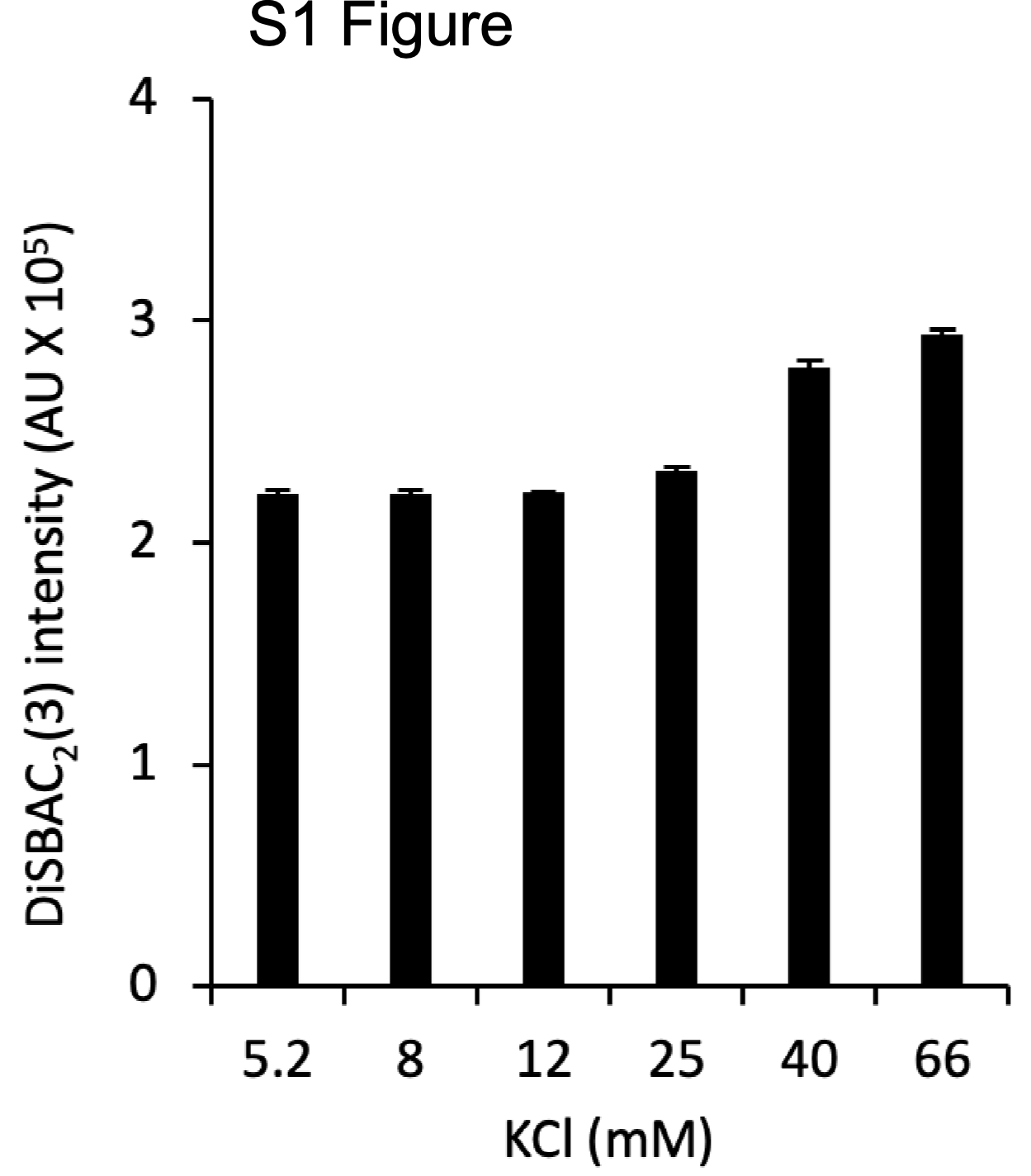

Supplement: S1 Fig — Sperm were incubated with 0–100 μM SFK inhibitors and then treated with DiSBAC2(3) for membrane polarization assays via measuring fluorescent intensity. Calibration was performed by adding 1 μM gramicidin and sequential additions of 1 M KCl solution to the medium (20 mM TES, 150 mM choline chloride, and pH 7.4), in which Na+ was replaced with choline+ at equal concentration. The initial potassium concentration was 5.2 mM KCl; additional amounts of KCl were added to the final concentration of 8, 12, 25, 40, and 66 mM KCl, corresponding to plasma Em of −80, −69, −59, −40, −28, and −15 mV, respectively, based on the Nernst equation (n = 8). (TIF) [file pone.0241181.s001.tif]

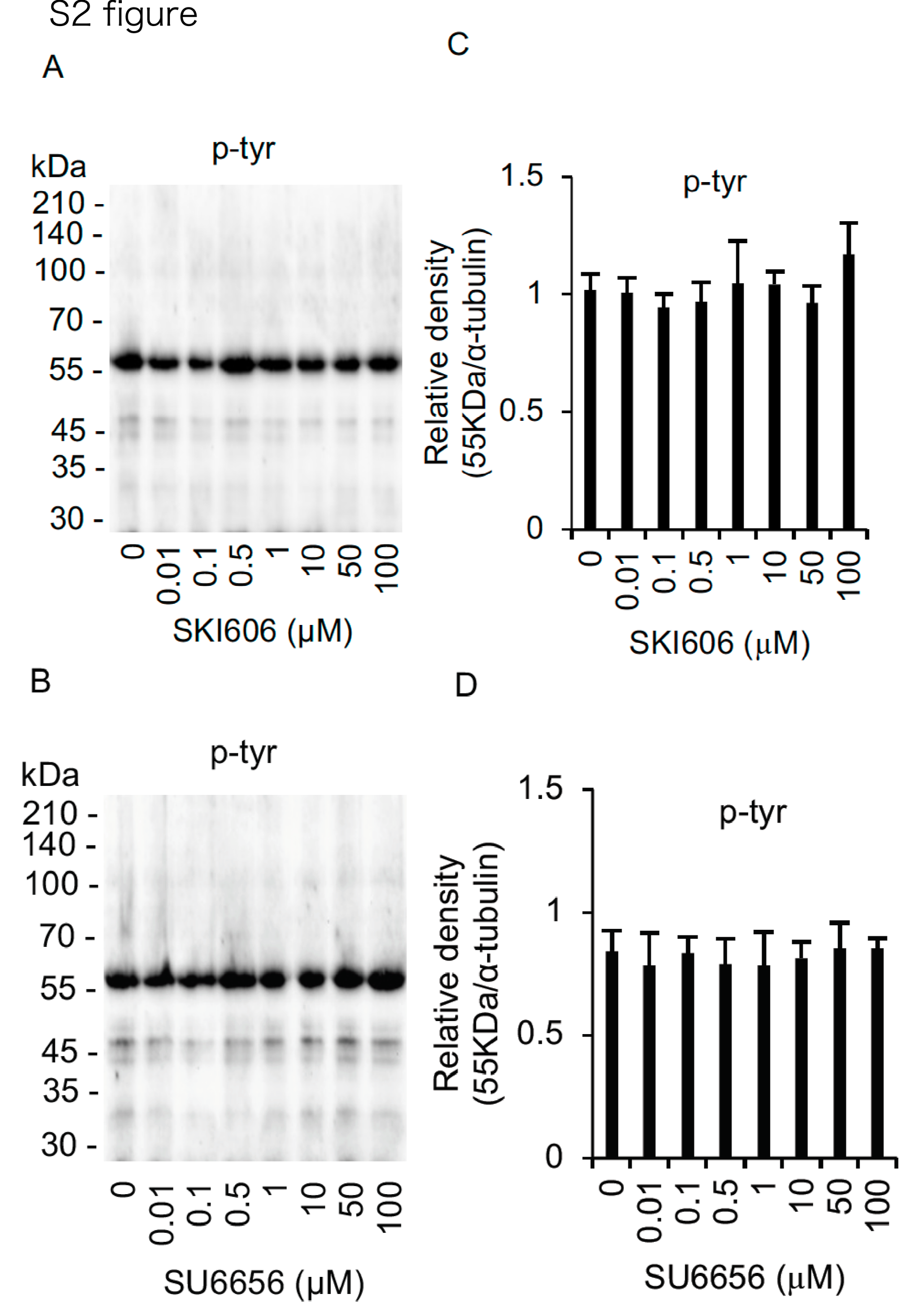

Supplement: S2 Fig — Sperm were incubated with 0–100 μM SKI606 or SU6656 and then subjected to immunoblotting. No difference was detected in p-tyrosine profile regardless of SFK inhibition by SKI606 (A and B). This is consistent with densitometry data (C and D). All immunoblotting analyses are representative of four replicate trials. Data are presented as mean ± SEM. (TIF) [file pone.0241181.s002.tif]

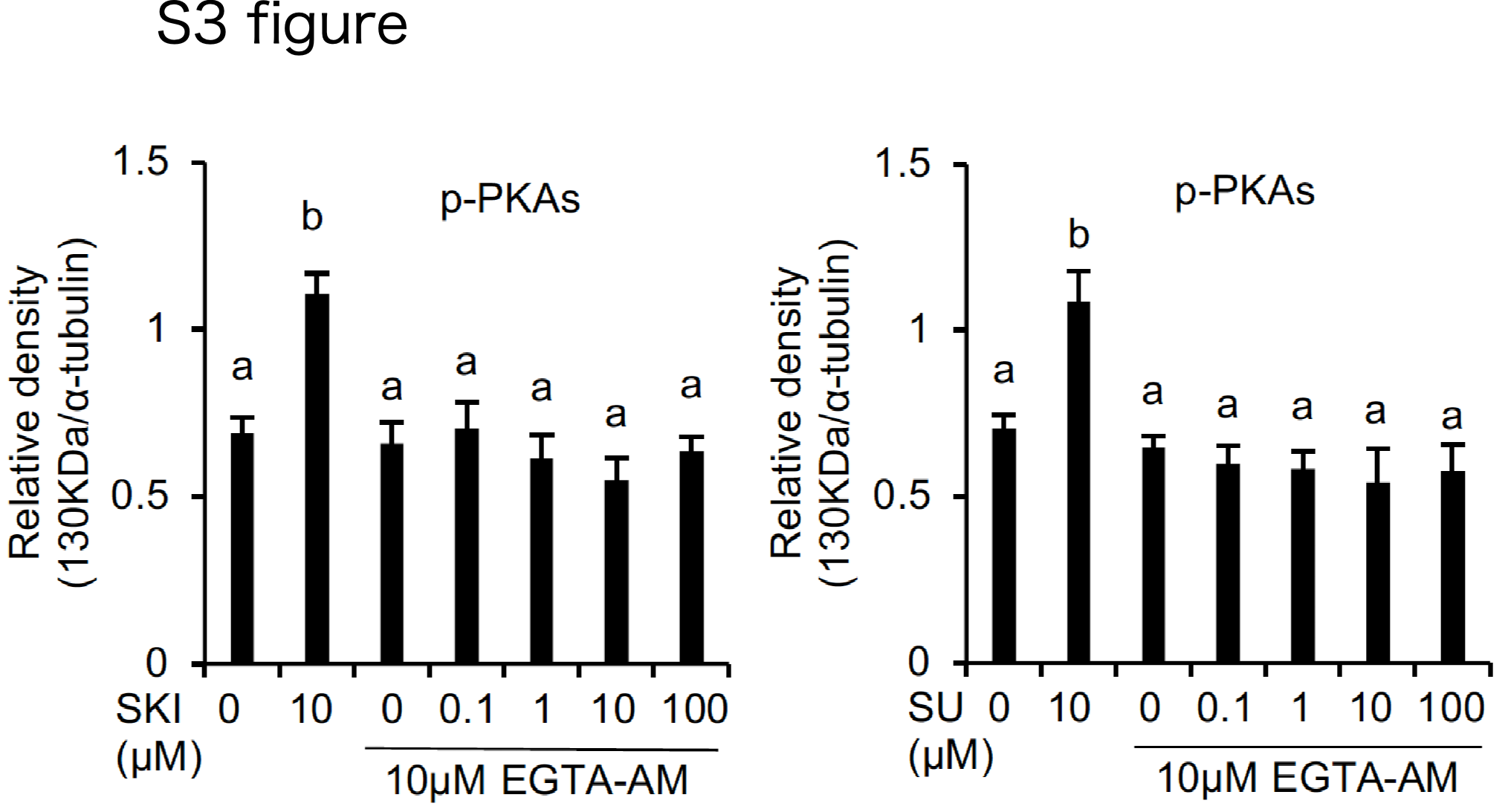

Supplement: S3 Fig — Sperm were preloaded with EGTA-AM for 15 min, and incubated under respective condition for 45 min. abP < 0.05. (TIF) [file pone.0241181.s003.tif]

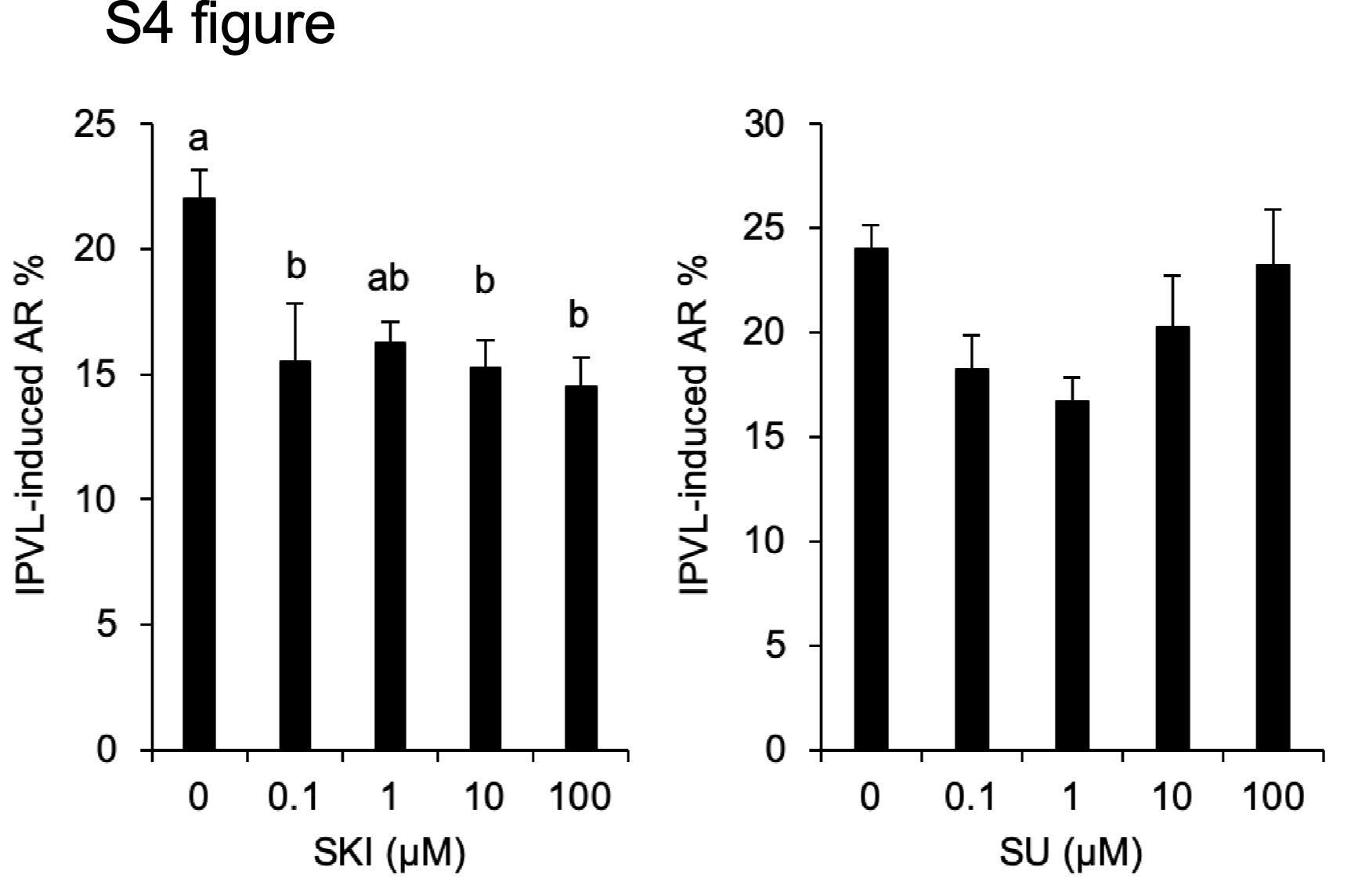

Supplement: S4 Fig — Net % of IPVL was obtained by subtracting of % spontaneous AR from total AR % after IPVL treatment. abP < 0.05. (TIF) [file pone.0241181.s004.tif]

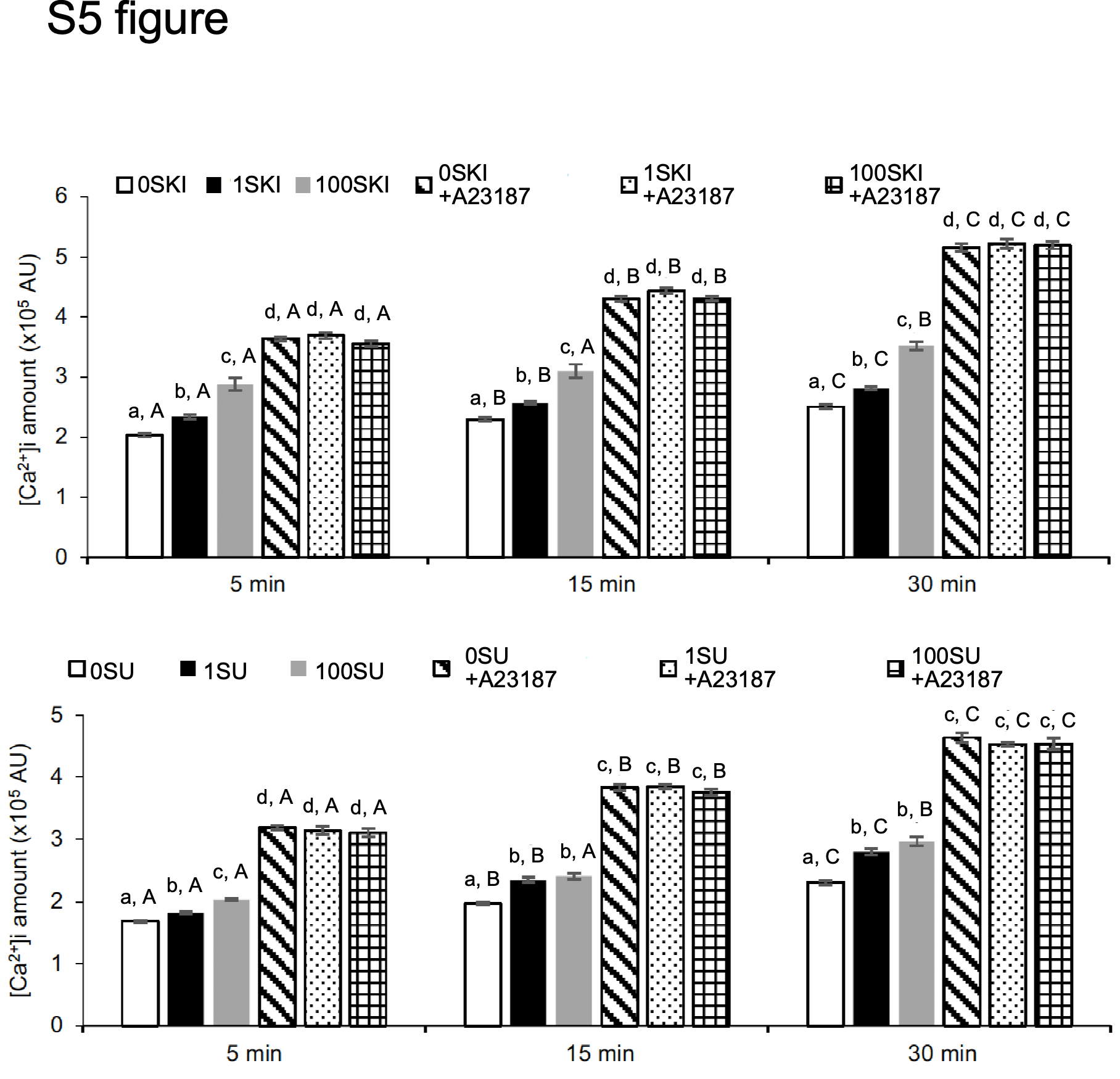

Supplement: S5 Fig — Sperm were loaded with Fluo 3-AM for 30 min. Fluorescence intensity of Fluo 3 was measured at 5, 15, and 30 min after incubation under the presence of 0, 1, and 100 μM SKI606 (SKI) or SU6656 (SU). Sperm were treated with 1 μM calcium ionophore as a positive control. adP < 0.05 in the same time point. ACP < 0.05 in the same treatment. (TIF) [file pone.0241181.s005.tif]

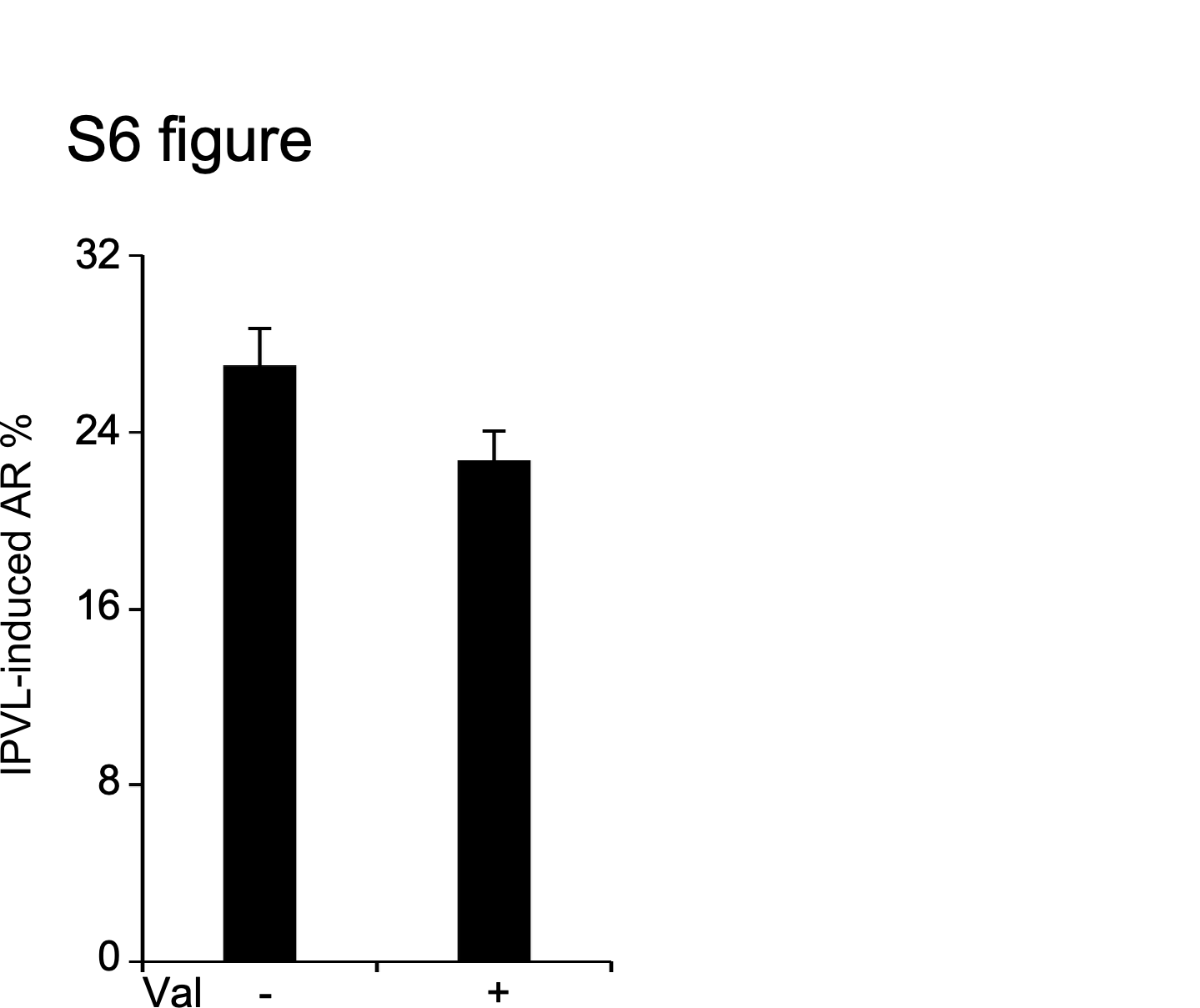

Supplement: S6 Fig — (TIF) [file pone.0241181.s006.tif]

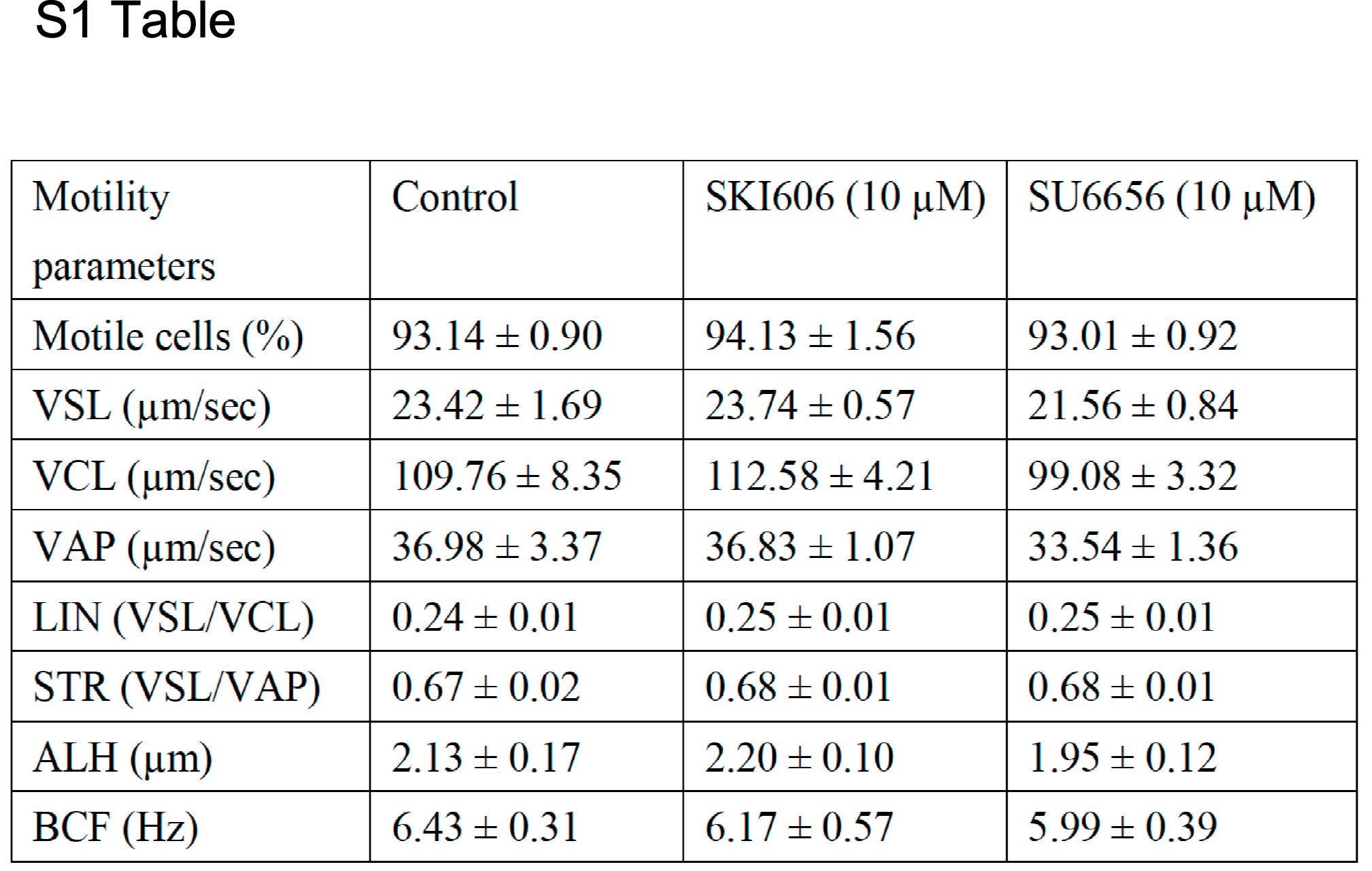

Supplement: S1 Table — Sperm were incubated with or without 10 μM SKI606 and SU6656 and were subjected to sperm motility analysis. No differences were observed in motility parameters across treatments. Data are presented as mean ± SEM (n = 4). (TIF) [file pone.0241181.s007.tif]
