# Supplementary material for: Src family kinases-mediated negative regulation of sperm acrosome reaction in chickens (Gallus gallus domesticus)
Source: PLoS One. 2020 Nov 12;15(11):e0241181. doi: 10.1371/journal.pone.0241181 (PMC7660528; doi:10.1371/journal.pone.0241181)
Supplement: S1 Raw images — (PDF) [file pone.0241181.s008.pdf]

Method for image-capturing

Following by western blotting, protein bands were visualized by enhanced chemiluminescence (Clarity ECL Substrate, Bio-Rad). The blot image was captured by ChemiDoc XRS+ system (Bio-Rad) using multiple exposures over the time where the timing is not consistent between images because of the avoidance of signal saturation.

Fig 1

A

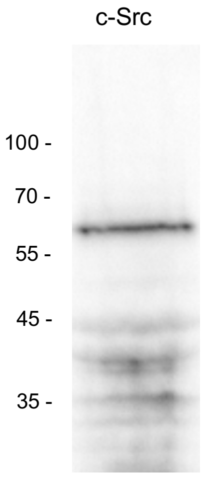

Fig 2

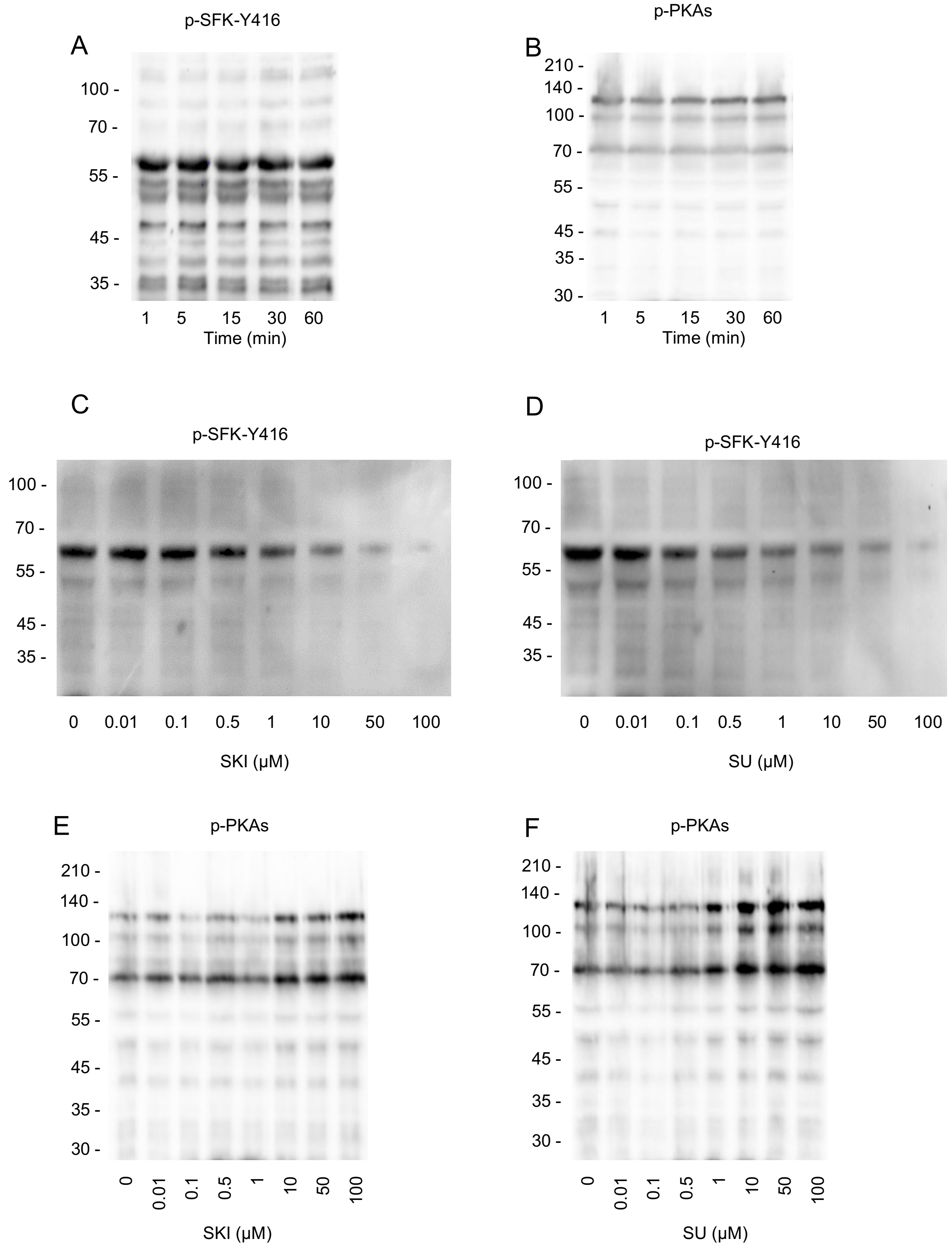

A
p-SFK-Y416
100 -
70 -
55 -
45 -
35 -
1 5 15 30 60
Time (min)
B
p-PKAs
210 -
140 -
100 -
70 -
55 -
45 -
35 -
30 -
1 5 15 30 60
Time (min)
C
p-SFK-Y416
100 -
70 -
55 -
45 -
35 -
0 0.01 0.1 0.5 1 10 50 100
SKI (μM)
D
p-SFK-Y416
100 -
70 -
55 -
45 -
35 -
0 0.01 0.1 0.5 1 10 50 100
SU (μM)
E
p-PKAs
210 -
140 -
100 -
70 -
55 -
45 -
35 -
30 -
0 0.01 0.1 0.5 1 10 50 100
SKI (μM)
F
p-PKAs
210 -
140 -
100 -
70 -
55 -
45 -
35 -
30 -
0 0.01 0.1 0.5 1 10 50 100
SU (μM)

Fig 3

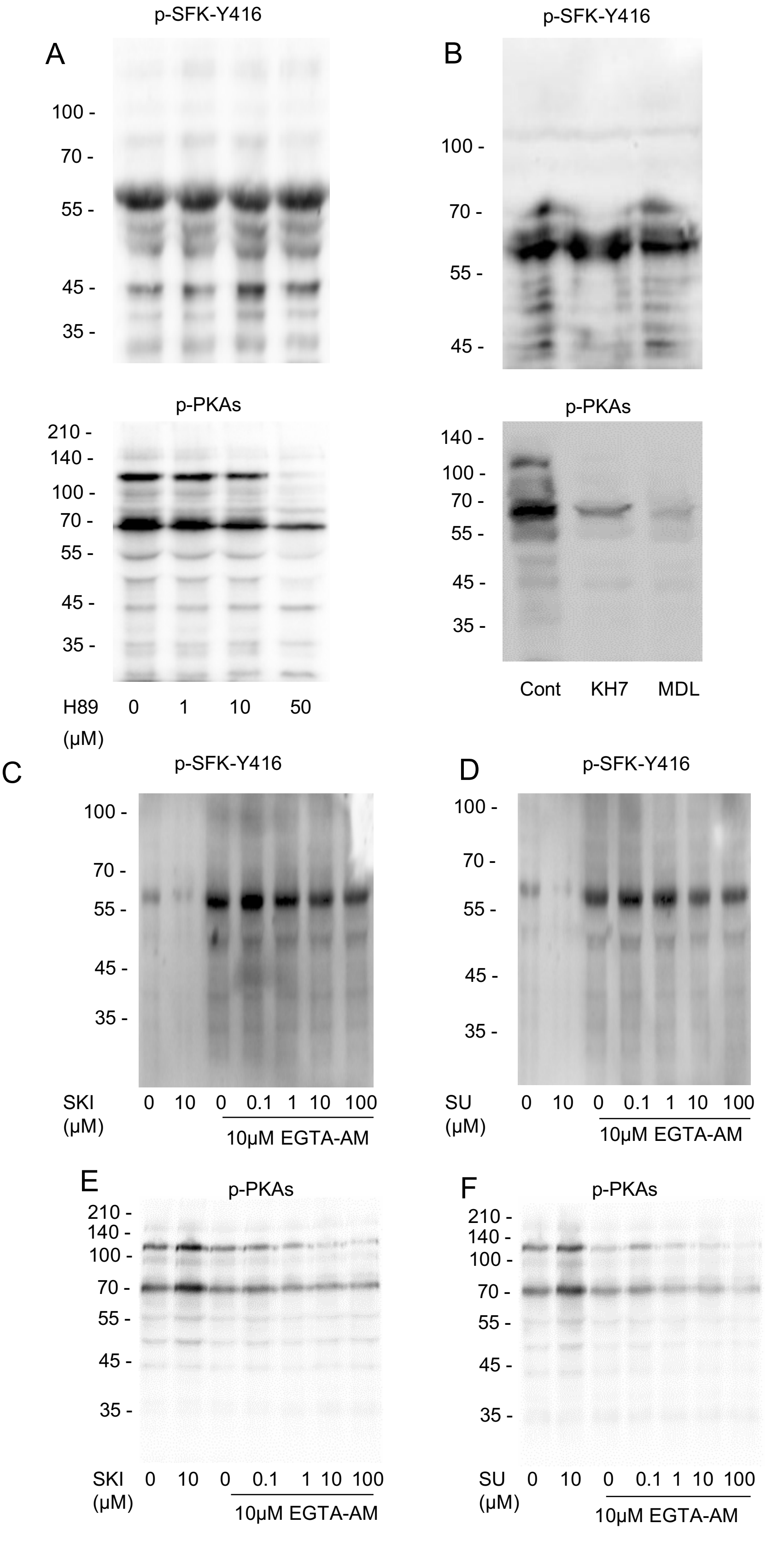
A
p-SFK-Y416
100 -
70 -
55 -
45 -
35 -
p-PKAs
210 -
140 -
100 -
70 -
55 -
45 -
35 -
H89 0 1 10 50
(μM)
B
p-SFK-Y416
100 -
70 -
55 -
45 -
p-PKAs
140 -
100 -
70 -
55 -
45 -
35 -
Cont KH7 MDL
C
p-SFK-Y416
100 -
70 -
55 -
45 -
35 -
SKI 0 10 0 0.1 1 10 100
(μM)
10μM EGTA-AM
D
p-SFK-Y416
100 -
70 -
55 -
45 -
35 -
SU 0 10 0 0.1 1 10 100
(μM)
10μM EGTA-AM
E
p-PKAs
210 -
140 -
100 -
70 -
55 -
45 -
35 -
SKI 0 10 0 0.1 1 10 100
(μM)
10μM EGTA-AM
F
p-PKAs
210 -
140 -
100 -
70 -
55 -
45 -
35 -
SU 0 10 0 0.1 1 10 100
(μM)
10μM EGTA-AM

Fig 3

G

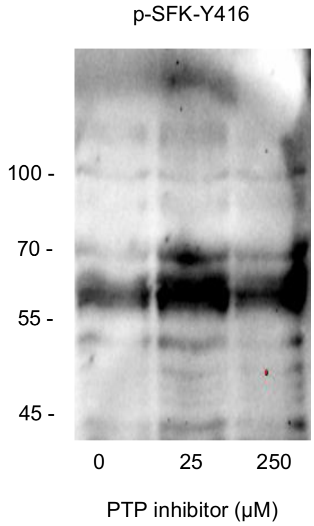
p-SFK-Y416
100 -
70 -
55 -
45 -
0
25
250
PTP inhibitor (μM)

H

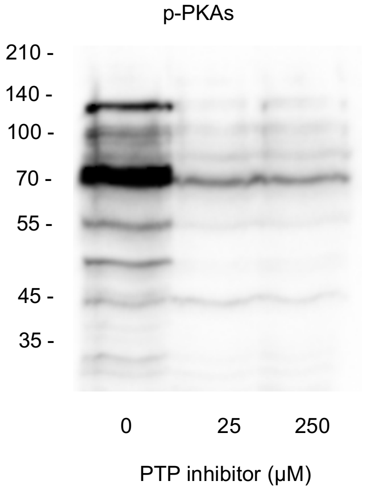
p-PKAs
210 -
140 -
100 -
70 -
55 -
45 -
35 -
0
25
250
PTP inhibitor (μM)

Fig 5

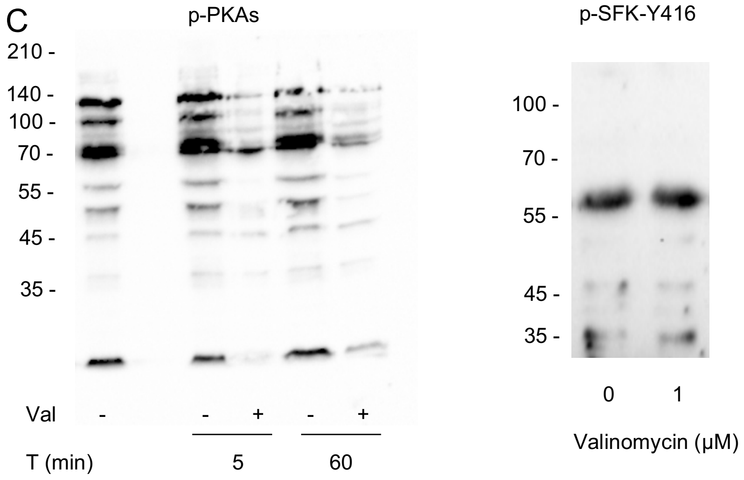
C
p-PKAs
210 -
140 -
100 -
70 -
55 -
45 -
35 -
Val - - + - +
T (min) 5 60
p-SFK-Y416
100 -
70 -
55 -
45 -
35 -
0 1
Valinomycin (μM)

Fig 6

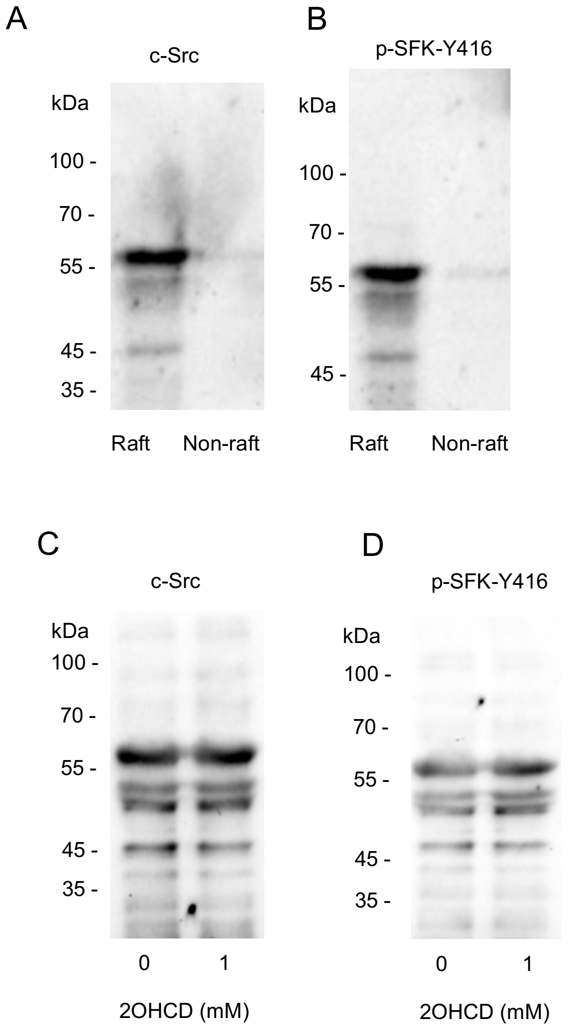
A
c-Src
kDa
100 -
70 -
55 -
45 -
35 -
Raft
Non-raft
B
p-SFK-Y416
kDa
100 -
70 -
55 -
45 -
Raft
Non-raft
C
c-Src
kDa
100 -
70 -
55 -
45 -
35 -
0
1
2OHCD (mM)
D
p-SFK-Y416
kDa
100 -
70 -
55 -
45 -
35 -
0
1
2OHCD (mM)
